# Factors influencing place of delivery: Evidence from three south-Asian countries

**DOI:** 10.1371/journal.pone.0250012

**Published:** 2021-04-08

**Authors:** Md. Ashfikur Rahman, Muhammad Aziz Rahman, Lal B. Rawal, Mohan Paudel, Md. Hasan Howlader, Bayezid Khan, Tanjim Siddiquee, Abdur Rahman, Apurbo Sarkar, Md. Sazedur Rahman, Roslin Botlero, Sheikh Mohammed Shariful Islam

**Affiliations:** 1 Development Studies Discipline, Khulna University, Khulna, Bangladesh; 2 School of Health, Federation University Australia, Berwick, VIC, Australia; 3 Australian Institute of Primary Care & Ageing (AIPCA), La Trobe University, Melbourne, VIC, Australia; 4 Department of Non-Communicable Diseases, Bangladesh University of Health Sciences (BUHS), Dhaka, Bangladesh; 5 School of Health Medical and Applied Sciences, College of Sciences and Sustainability, Central Queensland University, Sydney Campus, Sydney, Australia; 6 Adara Development Australia, Sydney, Australia; 7 Statistics Discipline, Khulna University, Khulna, Bangladesh; 8 Northwest A&F University, Yangling, China; 9 Monash Global and Women’s Health Unit, Monash University, Melbourne, VIC, Australia; 10 Institute for Physical Activity and Nutrition, Deakin University, Burwood, VIC, Australia; The University of Sydney, AUSTRALIA

## Abstract

**Background:**

High maternal mortality is still a significant public health challenge in many countries of the South-Asian region. The majority of maternal deaths occur due to pregnancy and delivery-related complications, which can mostly be prevented by safe facility delivery. Due to the paucity of existing evidence, our study aimed to examine the factors associated with place of delivery, including women’s preferences for such in three selected South-Asian countries.

**Methods:**

We extracted data from the most recent demographic and health surveys (DHS) conducted in Bangladesh (2014), Nepal (2016), and Pakistan (2017–18) and analyzed to identify the association between the outcome variable and socio-demographic characteristics. A total of 16,429 women from Bangladesh (4278; mean age 24.57 years), Nepal (3962; mean age 26.35 years), and Pakistan (8189; mean age 29.57 years) were included in this study. Following descriptive analyses, bivariate and multivariate logistic regressions were conducted.

**Results:**

Overall, the prevalence of facility-based delivery was 40%, 62%, and 69% in Bangladesh, Nepal, and Pakistan, respectively. Inequity in utilizing facility-based delivery was observed for women in the highest wealth quintile. Participants from Urban areas, educated, middle and upper household economic status, and with high antenatal care (ANC) visits were significantly associated with facility-based delivery in all three countries. Interestingly, watching TV was also found as a strong determinant for facility-based delivery in Bangladesh (aOR = 1.31, 95% CI:1.09–1.56, P = 0.003), Nepal (aOR = 1.42, 95% CI:1.20–1.67, P<0.001) and Pakistan (aOR = 1.17, 95% CI: 1.03–1.32, P = 0.013). Higher education of husband was a significant predictor for facility delivery in Bangladesh (aOR = 1.73, 95% CI:1.27–2.35, P = 0.001) and Pakistan (aOR = 1.19, 95% CI: 0.99–1.43, P = 0.065); husband’s occupation was also a significant factor in Bangladesh (aOR = 1.30, 95% CI:1.04–1.61, P = 0.020) and Nepal (aOR = 1.26, 95% CI:1.01–1.58, P = 0.041).

**Conclusion:**

Our findings suggest that the educational status of both women and their husbands, household economic situation, and the number of ANC visits influenced the place of delivery. There is an urgent need to promote facility delivery by building more birthing facilities, training and deployment of skilled birth attendants in rural and hard-to-reach areas, ensuring compulsory female education for all women, encouraging more ANC visits, and providing financial incentives for facility deliveries. There is a need to promote facility delivery by encouraging health facility visits through utilizing social networks and continuing mass media campaigns. Ensuring adequate Government funding for free maternal and newborn health care and local community involvement is crucial for reducing maternal and neonatal mortality and achieving sustainable development goals in this region.

## Introduction

Globally an estimated 810 maternal deaths occur every day, which are primarily preventable [1]. In 2017, a total of 295,000 women died due to childbirth and pregnancy-related complications, mainly in low-and middle-income countries (LMICs). In 2017, South-Asia and Sub-Saharan Africa constituted 86% (254,000) of the total maternal deaths combined, while South-Asia alone accounted for around one-fifth (58,000) of the total deaths [1]. Low utilization of facility delivery and lack of skilled birth attendants (SBAs) during delivery are the main factors contributing to high maternal mortality in these regions [2].

The Millennium Development Goal (MDGs 1990–2015) highlighted the importance of reducing maternal and child mortality by 75%. By 2015, this has resulted in a significant reduction of maternal mortality rate (MMR) to 38% worldwide [1]. The Sustainable Development Goal (SDG) 3 targets to reduce MMR to less than 70 deaths per 100,000 live births by 2030. Setting this ambitious SDG target is an excellent call to all nations, yet there are unfinished agendas of the MDG era. The remarkable gains made during the MDG era were not equally distributed across the world.

Bangladesh, Nepal, and Pakistan achieved praiseworthy progress in the reductions of MMR in the past few decades. From 2010 to 2017, MMR declined to 173/100000 live births in Bangladesh, 186/100000 in Nepal, and 140/1000000 in Pakistan [3]. However, MMR rates in these countries are still very high compared to the rates seen in other LMICs worldwide. Most of these deaths occur due to delivery complications that are largely preventable by changing childbirth from home to a health facility [3–7].

Evidence from several studies suggests that most maternal deaths occur when mothers cannot receive skilled care to manage a hemorrhage, sepsis, unsafe abortion, obstructed labor, and hypertensive disorders at home birth [4, 5, 7]. Studies have shown that 35% of all antepartum causes, intrapartum and postpartum hemorrhage are due to unsafe home delivery practices [4–6]. Many women in LMICs, including South-Asian countries, are still not receiving skilled care during pregnancy and childbirth. About 80% of MMR in LMICs can be averted by using SBAs and health facility delivery [8, 9]. The utilization of facility-based delivery services, family planning, antenatal and postnatal care expediates reductions in maternal deaths [1, 4, 10].

A range of factors hinders the utilization of facility delivery and other services during the pregnancy-postnatal continuum in these countries. Studies have identified poor health-seeking behavior, weak health systems, low socioeconomic status, cultural and personal health beliefs, lack of availability of appropriate health services, high cost, long-distance, lack of transport facilities, poor quality of the treatments are some of the critical barriers for low utilization of health care services [8, 11]. In the South-Asian context, women having home delivery are more likely to be affected by the unsafe and unhygienic environments, which in turn put mothers’ and newborns’ lives at risk of threatening conditions [5].

Although robust evidence exists on facility delivery benefits, that is not always practiced in many LMICs in the South-Asian region. The overall facility delivery rates are increasing in Nepal, Bangladesh and Pakistan over the last few decades [12–14]. South Asian countries have implemented different health care packages for pregnant mothers to promote facility delivery [12–14]. However, little is known about the factors influencing the place of delivery including women’s preferences on the place of childbirth in these countries. Therefore, our study aimed to examine the factors associated with facility delivery including women’s choices for the place of their childbirth.

## Methods

### Data sources and sampling procedures

We analyzed data from the most recent Demographic and Health Surveys (DHS) in Bangladesh, Nepal and Pakistan (BDHS 2014; NDHS 2016; PDHS 2017–18). Demographic and Health Surveys are periodic surveys carried out across these countries to identify the health status of their population [12–14]. A DHS survey offers a detailed overview of the study population and status on maternal and child health including a range of other thematic areas of health care. The dataset is publicly available online for academic and research purposes. All the DHS survey protocols obtained ethical approvals from both Institutional Review Board and the country-specific review boards. A detailed description of the survey strategy, methodology, sampling and questionnaires can be found in the final reports of these three countries [12–14].

### Outcome variable

Place of delivery (0 = Home, 1 = Facility) was the outcome variable in our analyses. The place of delivery was considered ’facility’ if a woman gave birth in a government hospital, district hospital, maternal and child welfare center (MCWC), Upazila health complex, health and family welfare center, private hospital/clinic, private medical college/hospital, rural health center, basic health unit, primary health care center and outreach clinic, or in a clinic run by family planning association. It was considered ’home delivery’ if a woman gave birth at the respondent’s own or relative’s/neighbor’s home.

### Predictor variables

A systematic literature search was performed to identify the predictor variables. This included review of most recent qualitative and quantitative studies from LMICs such as Bangladesh [15–18], Nepal [6, 7, 19–21], Pakistan [10], India [22–24], Eretria [9, 25], Tanzania [26], and sub-Sharan African countries [4, 27–29]. Table 1 provides a list of predictor variables identified from this review that could influence the place of delivery.

**Table 1:** Description of the variables.

SL.No.	Variables	Construction
**1**	Place of Residence	Rural*, Urban
**2**	Age of the Mother	15–24*, 25–34, 35–49
**3**	Mother’s BMI	Underweight*, Normal, Overweight/Obese
**4**	Mother’s Educational Level	No education*, Primary, Secondary, Higher
**5**	Mother’s Occupations	Working, Not Working*
**6**	Number of ANC Visits	Nil*, 1–3, ≥4
**7**	Total Number of Ever Born Child	1–2*, 3–4, ≥5
**8**	Ever had a Terminated Pregnancy	Yes, No*
**9**	Decision-Making Power on Delivery Place	Self, Both (Wife & Husband), Husband Alone, Someone Else*
**10**	Watching-TV	Yes, No*
**11**	Husband’s Education	No education*, Primary, Secondary, Higher
**12**	Husband’s Occupation	Agricultural*, Professional/Services, Others
**13**	Household Wealth Quantile	Poor*, Middle, Rich
**14**	Sources of Drinking Water	Improved Water, Non-improved Water*
**15**	Household Toilet Facility	Hygienic Toilet, Unhygienic Toilet*

*Asterisk stand for the Reference category

### Statistical analysis

The study considered the outcome and all the predictor variables as categorical data for the analyses. Descriptive statistics were used to summarize the background characteristics of the study population. Chi-square tests were performed to determine the association between the predictor variable and the place of delivery. Logistic regression analyses were conducted to determine the strength of associations by calculating odds ratios (ORs) and their 95% confidence intervals (CIs). The multivariate logistic regression was performed to examine the net effect of predictor variables on the outcome variable (facility delivery vs. home delivery) after adjusting for confounding factors. The statistically significant variables at p<0.05 level in the adjusted model are presented in the results section. Both unadjusted/crude (cOR) and adjusted odds ratios (aOR) have been reported in this paper.

### Ethical approval

The current study used publicly available data sources which already received ethical approvals for the primary studies in each country, thus did not require further ethical approval. The ethical consent was taken from the respective participants. The details of ethical procedures followed by the DHS program can be found elsewhere [26].

## Results

### Participants’ socio-demographic characteristics and place of delivery

Table 2 provides the prevalence of place of delivery by the participants’ socio-demographic characteristics. Overall, 40%, 62% and 69% of women gave birth at facilities in Bangladesh, Nepal and Pakistan, respectively. In Bangladesh, women residing in urban areas (53.5%), women with secondary education (53.5%), belonging to rich wealth quantile (62.3%), working women (80.7%) and those who had ≥4 ANC (49.6%) visits used health facilities for delivery. Younger women (15–24 years, 54.7%) used facility delivery. In Nepal, urban residents (41.7%), women aged 25–34 years (29.7%), having secondary education (25.6%), from high-income households (24.8%), working women (26.1%) and those who had ≥4 ANC visits (51.4%) had facility delivery. In Pakistan, urban residents (36.4%), belonging to rich wealth quantile (31.7%), those who had ≥4 ANC visits (62.3%), women aged 25–34 years (38.7%) used health facilities for delivery. The decision on the place of delivery or ANC visits during pregnancy was often made by the husband alone (27.0%).

**Table 2:** Percent distribution and preferred place of delivery by background characteristics: BDHS 2014, NDHS 2016, PDHS 2017–18.

Variables Level	Bangladesh 2014			Nepal 2016			Pakistan 2017		
	Place of Delivery			Place of Delivery			Place of Delivery		
	Facility (N/%)	Home (N/%)	P-Value	Facility (N/%)	Home (N/%)	P-Value	Facility (N/%)	Home (N/%)	P-Value
			χ2 test			χ2 test			χ2 test
**Place of Residence**			<0.001			<0.001			<0.001
Rural	913(31.5%)	1986(68.5%)		805(48.7%)	849(51.3%)		2733(60.7%)	1771(39.3%)	
Urban	793(57.5%)	586(42.5%)		1654(71.7%)	654(28.3%)		2977(80.8%)	708(19.2%)	
**Age of the Mother**			0.929			<0.001			<0.001
15–24	933(39.7%)	1417(60.1%)		1111(67.8%)	527(32.2%)		1320(71.0%)	539(29.0%)	
25–34	674(39.6%)	1002(60.4%)		1178(59.6%)	799(40.4%)		3167(71.6%)	1254(28.4%)	
35–49	99(62.2%)	153(37.8%)		170(49.0%)	177(51.0%)		1223(64.1%)	686(35.9%)	
**Mother’s BMI**			<0.001			<0.001			<0.001
Underweight	281(26.4%)	785(73.6%)		179(53.1%)	158(46.9%)		160(63.7%)	91(36.3%)	
Normal	1007(23.5%)	1533(35.8%)		2041(61.5%)	1277(38.5%)		4557(68.8%)	2064(31.2%)	
Overweight/Obese	418(62.2%)	254(37.8%)		239(77.9%)	68(22.1%)		993(75.4%)	324(24.6%)	
**Mother’s Educational Level**			<0.001			<0.001			<0.001
No education	102(17.9%)	467(82.1%)		495(40.8%)	719(59.2%)		2279(55.2%)	1852(44.8%)	
Primary	311(26.4%)	865(73.6%)		409(54.2%)	345(45.8%)		812(74.6%)	276(25.4%)	
Secondary	912(44.8%)	1125(55.2%)		1015(73.4%)	367(26.6%)		1458(84.2%)	273(15.8%)	
Higher	381(76.8%)	115(23.2%)		540(88.2%)	72(11.8%)		1161(93.7%)	78(6.3%)	
**Mother’s Occupations**			<0.001			<0.001			0.832
Working	330(33.2%)	663(66.8%)		1498(59.1%)	1036(40.9%)		757(69.4%)	333(30.6%)	
Not Working	1376(41.9%)	1909(58.1%)		961(67.3%)	467(32.7%)		4953(69.8%)	2146(30.2%)	
**Number of ANC Visits**			<0.001			<0.001			<0.001
Nil	92(10.1%)	820(89.9%)		47(19.4%)	195(80.6%)		338(26.6%)	932(73.4%)	
1–3	767(38.7%)	1217(61.3%)		377(39.7%)	572(60.3%)		1812(62.4%)	1091(37.6%)	
≥4	847(61.3%)	535(38.7%)		2035(73.4%)	736(26.6%)		3560(88.6%)	456(11.4%)	
**Total Number of Ever Born Child**			<0.001			<0.001			<0.001
1–2	1375(45.7%)	1635(54.3%)		1928(72.3%)	738(27.2%)		2593(78.8%)	697(21.2%)	
3–4	284(28.9%)	699(71.1%)		410(44.0%)	522(56.0%)		1862(69.9%)	801(30.1%)	
≥5	47(16.5%)	238(83.5%)		121(33.2%)	243(66.8%)		1255(56.1%)	981(43.9%)	
**Ever had a Terminated Pregnancy**			0.052			0.153			0.834
Yes	277(43.3%)	362(56.7%)		563(60.1%)	374(39.9%)		1723(69.9%)	741(30.1%)	
No	1429(39.3%)	2210(60.7%)		1896(62.7%)	1129(37.3%)		3987(69.7%)	1737(30.3%)	
**Decision-Making Power on Delivery Place**			0.003			<0.0012			<0.001
Self	203(41.1%)	291(58.9%)		550(64.9%)	297(35.1%)		501(78.2%)	140(21.8%)	
Both (Wife & Husband)	869(41.8%)	1209(58.2%)		735(64.0%)	413(36.0%)		2192(74.9%)	735(25.1%)	
Husband Alone	502(35.9%)	897(64.1%)		683(57.7%)	500(42.3%)		2212(63.4%)	1275(36.6%)	
Someone Else	132(43.0%)	175(57.0%)		491(62.6%)	293(37.4%)		805(71.0%)	329(29.0%)	
**Watching-TV**			<0.001			<0.001			<0.001
Yes	1310(56.0%)	1200(44.0%)		1792(72.8%)	670(27.2%)		3674(80.2%)	906(19.8%)	
No	396(24.4%)	1372(75.6%)		667(44.5%)	833(55.5%)		2036(56.4%)	1573(43.6%)	
**Husband’s Education**			<0.001			<0.001			<0.001
No education	196(20.1%)	778(79.9%)		201(39.6%)	307(60.4%)		1242(54.2%)	1049(45.8%)	
Primary	399(30.8%)	896(69.2%)		411(49.2%)	424(50.8%)		746(67.5%)	359(32.5%)	
Secondary	644(47.5%)	712(52.5%)		1240(66.5%)	625(33.5%)		2132(73.5%)	767(26.5%)	
Higher	467(71.5%)	186(28.5%)		607(80.5%)	146(19.5%)		1590(83.9%)	304(16.1%)	
**Husband’s Occupation**			<0.001			<0.001			<0.001
Agricultural	259(24.8%)	784(75.2%)		358(48.1%)	386(51.9%)		153(52.4%)	111(47.6%)	
Professional/Services	720(56.2%)	561(43.8%)		1135(73.9%)	400(26.1%)		333(74.7%)	68(25.3%)	
Others	727(37.2%)	1227(62.8%)		966(57.4%)	717(42.6%)		5224(88.7%)	2300(11.3%)	
**Household Wealth Quantile**			<0.001			<0.001			<0.001
Poor	345(20.1%)	1370(79.9%)		933(49.3%)	959(50.7%)		1915(52.4%)	1743(47.6%)	
Middle	298(36.2%)	526(63.8%)		453(68.5%)	250(31.5%)		1196(74.7%)	406(25.3%)	
Rich	1063(61.1%)	676(38.9%)		983(77.0%)	294(23.0%)		2599(88.7%)	330(11.3%)	
**Sources of Drinking Water**			<0.001			0.011			<0.001
Improved Water	1695(40.3%)	2506(59.7%)		2349(62.5%)	1408(37.5%)		5312(71.2%)	2148(28.8%)	
Non-improved Water	11(14.3%)	66(85.7%)		110(53.7%)	95(46.3%)		398(54.6%)	331(45.4%)	
**Household Toilet Facility**			<0.001			<0.001			<0.001
Hygienic Toilet	1473(46.9%)	1668(53.1%)		2221(66.0%)	1143(34.0%)		5135(73.1%)	1893(26.9%)	
Unhygienic Toilet	233(20.5%)	904(79.5%)		238(39.8%)	360(60.2%)		575(49.5%)	586(50.5%)	

Note: Except few all the independent variables are statistically significant with the dependent variables at P ⩽ 0.01, 0.01, P ⩽ 0.05, 0.05 P ⩽ 0.10 level. The insignificant variables are not adjusted in the final model.

In bivariate analysis, a statistically significant association was found between facility delivery and most of the predictor variables across all three countries, which are listed in Table 1. The regression analysis revealed a significant association between facility delivery and urban residing women, secondary and higher education level, middle and rich households and more ANC visits, watching TV, and husband involved in high income earning professions across all three countries. However, women’s age in Bangladesh and women’s education and history of abortion were not found statistically significant in Pakistan.

In Bangladesh (Table 3), factors associated with facility delivery were: residing in urban areas compared to residing in rural areas (aOR = 1.49, 95% CI:1.26–1.76, P<0.001), education level of women at primary (aOR = 1.80, 95% CI: 1.37–2.36, P<0.001), secondary (aOR = 2.17, 95% CI:1.58–2.99; P<0.001) and higher level (aOR = 2.28, 95% CI:1.54–3.37, P<0.001) compared to those women who had no education. Women who had ≥4 ANC visits during pregnancy (aOR = 5.64, 95% CI:4.34–7.32), P<0.001) were more likely to use facility delivery compared to women with less or no ANC visits. Women who reported watching TV (aOR = 1.31, 95% CI:1.09–1.56, P = 0.003) compared with those not watching TV, who had husbands with secondary (aOR = 1.26, 95% CI:1.00–1.60, P = 0.054) or higher education level (aOR = 1.73, 95% CI:1.27–2.35, P = 0.001), husband with high-income profession (aOR = 1.30, 95% CI:1.04–1.61, P = 0.020), whose households belonged to middle income family (aOR = 1.23, 95% CI:0.99–1.52, P = 0.059) or high-income family (1.66, 95% CI:1.34–2.07, P<0.001), and those having access to hygienic toilet at home (aOR = 1.32, 95% CI:1.09–1.60, P = 0.005) were more likely to have facility delivery than those who did not have hygienic toilet. On the other hand, factors associated with low use of facility delivery were working women (aOR = 0.77, 95% CI:0.65–0.923, P = 0.004), and women with 3 to 4 children (aOR = 0.69, 95% CI:0.57–0.83, P<0.001).

**Table 3:** Regression results factors associated with health facility delivery by background characteristics: BDHS 2014.

Variables Level	Facility (N/%)	Home (N/%)	cOR	P-Value	aOR	P-Value
	1706	2572				
**Place of Residence**						
Rural	913(53.5)	1986(77.2)	1		1	<0.001
Urban	793(46.5)	586(22.8)	2.94(2.58–3.36)	<0.001	1.49(1.26–1.76)	
**Age of the Women**						
15–24	933(54.7)	1417(55.1)	-		-	
25–34	674(39.5)	1002(39.0)	-		-	
35–49	99(5.8)	153(5.9)	-		-	
**Women’s BMI**						
Underweight	281(16.5)	785(30.5)	1		1	
Normal	1007(59.0)	1533(59.6)	1.84(1.57–2.15)	<0.001	1.37(1.14–1.64)	0.001
Overweight/Obese	418(24.5)	254(9.9)	4.60(3.74–5.66)	<0.001	2.22(1.74–2.83)	<0.001
**Women’s Educational Level**						
No education	102(6.0)	467(18.2)	1		1	
Primary	311(18.2)	865(33.6)	1.65(1.28–2.11)	<0.001	1.80(1.37–2.36)	<0.001
Secondary	912(53.5)	1125(43.7)	3.71(2.95–4.68)	<0.001	2.17(1.58–2.99)	<0.001
Higher	381(22.3)	115(4.5)	15.2(11.3–20.5)	<0.001	2.28(1.54–3.37)	<0.001
**Women’s Occupations**						
Not Working	330(19.3)	663(25.8)	1		1	
Working	1376(80.7)	1909(74.2)	0.69(0.60–0.80)	<0.001	0.77(0.65–0.923)	0.004
**Number of ANC Visits**						
Nil	92(5.4)	820(31.9)	1		1	
1–3	767(45.0)	1217(47.3)	5.62(4.45–7.10)	<0.001	3.33(2.60–4.27)	<0.001
≥4	847(49.6)	535(20.8)	14.1(11.9–18.0)	<0.001	5.64(4.34–7.32)	<0.001
**Total Number of Ever Born Child**						
1–2	1375(80.6)	1635(63.6)	1		1	
3–4	284(16.6)	699(27.2)	0.48(0.41–0.56)	<0.001	0.69(0.57–0.83)	<0.001
≥5	47(2.8)	238(5.6)	0.24(0.17–0.32)	<0.001	0.60(0.41–0.87)	0.008
**Ever had a Terminated Pregnancy**						
No	277(16.2)	362(14.1)	1		1	
Yes	1429(83.8)	2210(85.9)	1.18(1.00–1.40)	0.052	1.08(0.884–1.32)	0.449
**Decision Making Power on Respondent’s Health Care**						
Someone Else	203(11.9)	291(11.3)	1		1	
Self	869(50.9)	1209(47.0)	0.93(0.69–1.23)	0.595	0.95(0.68–1.33)	0.782
Both (Wife & Husband)	502(29.4)	897(34.9)	0.95(0.75–1.21)	0.696	1.09(0.83–1.45)	0.536
Husband Alone	132(7.7)	175(6.8)	0.74(0.58–0.95)	0.020	1.04(0.78–1.39)	0.793
**Watching-TV**						
No	1310(76.8)	1200(46.7)	1		1	
Yes	396(23.2)	1372(53.3)	3.78(3.30–4.34)	<0.001	1.31(1.09–1.56)	0.003
**Husband’s Education**						
No education	196(11.5)	778(30.2)	1		1	
Primary	399(23.4)	896(34.8)	1.77(1.45–2.15)	<0.001	1.17(0.94–1.45)	0.162
Secondary	644(37.7)	712(27.7)	3.59–2.97–4.34)	<0.001	1.26(1.00–1.60)	0.054
Higher	467(27.4)	186(7.2)	9.97(7.91–12.6)	<0.001	1.73(1.27–2.35)	0.001
**Husband’s Occupation**						
Agricultural	259(15.2)	784(30.5)	1		1	
Professional/Services	720(42.2)	561(21.8)	3.89(3.25–4.65)	<0.001	1.30(1.04–1.61)	0.020
Others	727(42.6)	1227(47.7)	1.79(1.52–2.12)	<0.001	0.93(0.76–1.23)	0.440
**Household Wealth Quantile**						
Poor	345(20.2)	1370(53.3)	1		1	
Middle	298(17.5)	526(20.5)	2.25(1.87–2.71)	<0.001	1.23(0.99–1.52)	0.059
Rich	1063(62.3)	676(26.3)	6.24(5.36–7.27)	<0.001	1.66(1.34–2.07)	<0.001
**Sources of Drinking Water**						
Improved Water	11(0.6)	66(2.6)	1		1	
Non-improved Water	1695(99.4)	2506(97.4)	4.06(2.14–7.71)	<0.001	1.30(0.64–2.65)	0.466
**Household Toilet Facility**						
Unhygienic Toilet	233(13.7)	904(35.1)	1		1	
Hygienic Toilet	1473(86.3)	1668(64.9)	3.43(2.92–4.02)	<0.001	1.32(1.09–1.60)	0.005

In Nepal (Table 4), women living in urban areas compared to rural areas (aOR = 2.17, 95% CI:1.85–2.54, P<0.001), aged 35–49 years (aOR = 1.43, 95% CI:1.01–2.03, P = 0.044), being overweight (aOR = 1.60, 95% CI:1.07–2.41, P = 0.023), having secondary and higher education (aOR = 1.55, 95% CI:1.23–1.94, P = <0.001) and (aOR = 2.56, 95% CI:1.80–3.64, P<0.001) respectively, were more likely to use facility delivery as compared to their counterparts living in rural areas, aged (25–34 years), with normal BMI, women with no education. Women who had ≥4 ANC visits compared to those women who had not visited ANC (aOR = 5.48, 95% CI:3.84–7.82, P<0.001), women who reported watching TV (aOR = 1.42, 95% CI:1.20–1.67,P<0.001) compared with those not watching TV, whose husbands were involved in high income profession (aOR = 1.26, 95% CI:1.01–1.58,P = 0.041) compared to the husbands involved in agriculture, households in the middle-income family (aOR = 1.61, 95% CI:1.31–1.98, P<0.001) and high-income family (aOR = 2.32, 95% CI:1.88–2.86, P<0.001) than poor-income family, and households with hygienic toilets (aOR = 1.41, 95% CI:1.14–1.74, P = 0.002) were more likely to use facility delivery than those without hygienic toilet. On the other hand, factors associated with low use of facility delivery were: working women (aOR = 0.85, 95% CI: 0.72–1.01, P = 0.069), women with 3 to 4 children (aOR = 0.53, 95% CI: 0.44–0.65,P<0.001) and having children ≥5 (aOR = 0.47, 95% CI:0.34–0.65, P<0.001). On the other hand, factors associated with low use of facility delivery were: working women (aOR = 0.85, 95% CI: 0.72–1.01, P = 0.069), women with 3 to 4 children (aOR = 0.53, 95% CI: 0.44–0.65,P<0.001) and having children ≥5 (aOR = 0.47, 95% CI:0.34–0.65, P<0.001).

**Table 4:** Regression results factors associated with health facility delivery by background characteristics: NDHS 2016.

Variables Level	Facility (N/%)	Home (N/%)	cOR	P-Value	aOR	P-Value
	1706	2572				
**Place of Residence**						
Rural	1654(67.3)	654(43.5)	1		1	
Urban	805(32.7)	849(56.5)	2.67(2.34–3.04)	<0.001	2.17(1.85–2.54)	<0.001
**Age of the Women**						
15–24	1111(45.2)	527(35.1)	1		1	
25–34	1178(47.9)	799(53.2)	0.70(0.61–0.80)	<0.001	0.91(0.76–1.10)	0.321
35–49	170(6.9)	177(11.8)	0.46(0.36–0.58)	<0.001	1.43(1.01–2.03)	0.044
**Women’s BMI**						
Underweight	179(7.3)	158(10.5)	1		1	
Normal	2041(83.0)	1277(85.0)	1.41(1.13–1.77)	0.003	1.12(0.86–1.46)	0.390
Overweight/Obese	239(9.7)	68(4.5)	3.10(2.20–4.38)	<0.001	1.60(1.07–2.41)	0.023
**Mother’s Educational Level**						
No education	495(20.1)	719(47.8)	1		1	
Primary	409(16.6)	345(23.0)	1.72(1.43–2.07)	<0.001	1.14(0.92–1.42)	0.237
Secondary	1015(41.3)	367(24.4)	4.02(3.41–4.74)	<0.001	1.55(1.23–1.94)	<0.001
Higher	540(22.0)	72(4.8)	10.9(8.30–14.3)	<0.001	2.56(1.80–3.64)	<0.001
**Mother’s Occupations**						
Not Working	1498(60.9)	1036(68.9)	1		1	
Working	961(39.1)	467(31.1)	0.70(0.61–0.81)	<0.001	0.85(0.72–1.01)	0.069
**Number of ANC Visits**						
Nil	47(1.9)	195(13.0)	1		1	
1–3	377(15.3)	572(38.1)	2.74(1.94–3.86)	<0.001	2.06(1.42–2.98)	<0.001
≥4	2035(82.8)	736(49.0)	11.5(8.25–15.9)	<0.001	5.48(3.84–7.82)	<0.001
**Total Number of Ever Born Child**						
1–2	1928(78.4)	738(49.1)	1		1	
3–4	410(16.7)	522(34.7)	0.30(0.26–0.35)	<0.001	0.53(0.44–0.65)	<0.001
≥5	121(4.9)	243(16.2)	0.19(0.15–0.24)	<0.001	0.47(0.34–0.65)	<0.001
**Ever had a Terminated Pregnancy**						
No	563(22.9)	374(24.9)	1		1	
Yes	1896(77.1)	1129(75.1)	0.90(0.77–1.04)	0.153	1.06(0.89–1.27)	0.528
**Decision Making Power on Respondent’s Health Care**						
Someone Else	491(20.0)	293(7.4)	1		1	
Self	550(22.4)	297(19.8)	1.11(0.90–1.35)	0.333	1.10(0.86–1.40)	0.451
Both (Wife & Husband)	735(29.9)	413(27.5)	1.06(0.88–1.28)	0.531	1.04(0.83–1.30)	0.734
Husband Alone	683(27.8)	500(33.3)	0.82(0.68–0.98)	0.030	1.08(0.86–1.34)	0.520
**Watching-TV**						
No	667(27.1)	833(55.4)	1		1	
Yes	1792(72.9)	670(44.6)	3.34(2.92–3.82)	<0.001	1.42(1.20–1.67)	<0.001
**Husband’s Education**						
No education	201(8.2)	307(20.4)	1		1	
Primary	411(16.7)	424(28.2)	1.48(1.18–1.85)	0.001	1.06(0.82–1.38)	0.656
Secondary	1240(50.4)	625(41.6)	3.03(2.48–3.71)	<0.001	1.12(0.87–1.44)	0.363
Higher	607(24.7)	146(9.8)	6.31(4.90–8.12)	<0.001	1.28(0.92–1.79)	0.145
**Husband’s Occupation**						
Agricultural	358(14.6)	386(25.7)	1		1	
Professional/Services	1135(46.2)	400(26.6)	3.06(2.55–3.68)	<0.001	1.26(1.01–1.58)	0.041
Others	966(39.3)	717(47.7)	1.45(1.22–1.73)	<0.001	1.09(0.88–1.34)	0.438
**Household Wealth Quantile**						
Poor	933(37.9)	959(63.8)	1		1	
Middle	453(22.1)	250(16.6)	2.23(1.87–2.66)	<0.001	1.61(1.31–1.98)	<0.001
Rich	983(40.0)	294(19.6)	3.44(2.93–4.03)	<0.001	2.32(1.88–2.86)	<0.001
**Sources of Drinking Water**						
Improved Water	110(4.5)	95(6.3)	1		1	
Non-improved Water	2349(95.5)	1408(93.7)	1.44(1.09–1.91)	0.011	0.88(0.63–1.22)	0.429
**Household Toilet Facility**						
Unhygienic Toilet	238(9.7)	360(24.0)	1		1	
Hygienic Toilet	2221(90.3)	1143(76.0)	2.94(2.46–3.51)	<0.001	1.41(1.14–1.74)	0.002

In Pakistan (Table 5), women from urban areas compared to those who resided in rural areas (aOR = 1.21, 95% CI:10.7–138, P = 0.003), women having secondary (aOR = 1.44, 95% CI:1.21–1.70, P<0.001) or higher education (aOR = 2.83, 95% CI: 2.15–3.70, P<0.001) were statistically strongly associated with facility delivery compared to those who had no education. Women who had ≥4 times ANC visits compared to those women who had not seeking ANC (aOR = 10.24, 95% CI: 8.6–12.17, P<0.001), whose husbands had higher education (aOR = 1.19, 95% CI: 0.99–1.43, P = 0.065), watched TV (aOR = 1.17, 95% CI: 1.03–1.32, P = 0.013), from households belonged to middle income (aOR = 1.36, 95% CI:1.17–1.59, P<0.001) and rich income family (aOR = 1.83, 95% CI:1.54–2.18,P<0.001) were more likely to have facility delivery. On the other hand, factors associated with low use of facility delivery were: women with 3 to 4 children (aOR = 0.75, 95% CI:0.65–0.87, P<0.001) and having children ≥5 (aOR = 0.64, 95% CI: 0.54–0.76, P<0.001).

**Table 5:** Regression results factors associated with health facility delivery by background characteristics: PDHS 2017–18.

Variables Level	Facility (N/%)	Home (N/%)	cOR	P-Value	aOR	P-Value
	5710	2479				
**Place of Residence**						
Rural	2977(52.1)	708(28.6)	1		1	
Urban	2733(47.9)	1771(71.4)	2.60(2.35–2.87)	<0.001	1.21(10.7–138)	0.003
**Age of the Women**						
15–24	1320(23.1)	539(21.7)	1		1	
25–34	3167(55.5)	1254(50.6)	1.06(0.94–1.18)	0.410	1.06(0.91–1.24)	0.454
35–49	1223(21.4)	686(27.7)	0.77(0.67–0.88)	<0.001	1.20(0.98–1.46)	0.081
**Women’s BMI**						
Underweight	160(2.8)	91(3.7)	1		1	
Normal	4557(79.8)	2064(83.3)	1.28(0.99–1.66)	0.059	1.00(0.75–1.35)	0.980
Overweight/Obese	993(17.4)	324(13.1)	1.77(1.34–2.35)	<0.001	1.12(0.81–1.55)	0.509
**Women’s Educational Level**						
No education	2279(39.9)	1852(74.7)	1		1	
Primary	812(14.2)	276(11.1)	2.25(1.94–2.59)	<0.001	1.16(0.98–1.37)	0.082
Secondary	1458(25.5)	273(11.0)	4.09(3.57–4.69)	<0.001	1.44(1.21–1.70)	<0.001
Higher	1161(20.3)	78(3.1)	12.4(9.93–15.5)	<0.001	2.83(2.15–3.70)	<0.001
**Women’s Occupations**						
Working	757(13.3)	333(13.4)	-		-	
Not Working	4953(86.7)	2146(86.6)	-		-	
**Number of ANC Visits**						
Nil	338(5.9)	932(37.6)	1		1	
1–3	1812(31.7)	1091(44.0)	4.63(3.99–5.38)	<0.001	3.56(3.05–4.16)	<0.001
≥4	3560(62.3)	456(18.4)	21.2(18.1–24.9)	<0.001	10.24(8.6–12.17)	<0.001
**Total Number of Ever Born Child**						
1–2	2593(45.4)	697(28.1)	1		1	
3–4	1862(32.6)	801(32.3)	0.65(0.58–0.72)	<0.001	0.75(0.65–0.87)	<0.001
≥5	1255(22.0)	981(39.6)	0.36(0.32–0.40)	<0.001	0.64(0.54–0.76)	<0.001
**Ever had a Terminated Pregnancy**						
No	3987(69.8)	1737(70.1)	-		-	
Yes	1723(30.2)	741(29.9)	-		-	
**Decision Making Power on Respondent’s Health Care**						
Someone Else	805(14.1)	329(13.3)	1		1	
Self	501(8.8)	140(5.6)	1.42(1.14–1.77)	0.002	0.95(0.74–1.23)	0.703
Both (Wife & Husband)	2192(38.4)	735(29.6)	1.27(1.10–1.48)	0.001	1.02(0.85–1.22)	0.826
Husband Alone	2212(38.7)	1275(51.4)	0.73(0.64–0.85)	<0.001	0.88(0.74–1.04)	0.128
**Watching-TV**						
No	2036(35.7)	1573(63.5)	1		1	
Yes	3674(64.3)	906(36.5)	3.10(2.82–3.41)	<0.001	1.17(1.03–1.32)	0.013
**Husband’s Education**						
No education	1242(21.8)	1049(42.3)	1		1	
Primary	746(13.1)	359(14.5)	1.76(1.52–2.04)	<0.001	1.14(0.96–1.35)	0.137
Secondary	2132(37.3)	767(30.9)	2.40(2.13–2.68)	<0.001	1.04(0.90–1.20)	0.605
Higher	1590(27.8)	304(12.3)	4.51(3.90–5.20)	<0.001	1.19(0.99–1.43)	0.065
**Husband’s Occupation**						
Agricultural	153(2.7)	111(4.5)	1		1	
Professional/Services	333(5.8)	68(2.8)	4.29(3.02–6.08)	<0.001	0.86(0.57–1.29)	0.471
Others	5224(91.5)	2300(92.8)	1.97(1.54–2.53)	<0.001	0.98(0.74–1.30)	0.891
**Household Wealth Quantile**						
Poor	1915(33.5)	1743(70.3)	1		1	
Middle	1196(20.9)	406(16.4)	2.57(2.26–2.91)	<0.001	1.36(1.17–1.59)	<0.001
Rich	2599(45.5)	330(13.3)	6.69(5.90–7.58)	<0.001	1.83(1.54–2.18)	<0.001
**Sources of Drinking Water**						
Improved Water	398(7.0)	331(13.4)	1		1	
Non-improved Water	5312(93.0)	2148(86.6)	1.98(1.70–2.31)	<0.001	0.98(0.82–1.18)	0.840
**Household Toilet Facility**						
Unhygienic Toilet	575(10.1)	586(23.6)	1		1	
Hygienic Toilet	5135(89.9)	1893(76.4)	2.82(2.48–3.20)	<0.001	0.96(0.82–1.12)	0.580

## Discussion

This study examined factors associated with place of delivery using nationally representative surveys in three South-Asian countries. This study suggests that women from urban areas, having a secondary and higher level of education and higher education levels of their husbands, middle and upper-income households, women having higher ANC (≥4) visits, watching TVs and husbands with high-income families are found statistically significantly associated with increased facility delivery. These findings would be useful for the government and stakeholders for planning, designing and implementing appropriate interventions and addressing the barriers to improving utilization of health facilities, and thereby contributing to reducing maternal mortality in South-Asian countries.

In Bangladesh, consecutive demographic surveys (28.7% in 2011, 40% in 2014 and 50% in 2018) [30] indicated a rising trend in facility delivery. However, this trend is quite slow compared to the decline rates seen in Nepal, India and Pakistan in the same period [10, 15]. Health transition through social transformation [31] is considered as one key factor contributing to the increasing trend of facility delivery in Bangladesh. In Nepal, the facility delivery rate was quite low from 1996 till 2001 [14]. However, after five years, the delivery rate doubled to 18% in 2006 and quadrupled to 35% in 2011 [14]. In Pakistan, this trend increased from 34% in 2006–07 to 48% in 2012–13, and 69.4% in 2016–17 period [13]. The facility delivery rate is gradually increasing in this region which could be due to ongoing social mobilization and continuous supports from various NGOs [32] along with increased literacy and girls enrolment rates in schools, availability of better health care and family planning services in health facilities, access to community clinics, the introduction of free delivery services and maternity incentive schemes by governments. In addition, government maternity incentive schemes and subsidies increase the number of birthing facilities in rural areas and further encourage women to attend facility delivery [19, 31, 33, 34].

Our results are similar to previous studies, where high socio-economic status, higher education level of husband, urban residing women and more ANC visits had a significant influence on women to utilize facility delivery [9, 15, 19, 20, 25]. The plausible explanations could be urban women are more likely to be educated and hence more health-conscious and have better access to health care services than women from rural areas. This would lead to a regular and increased number of ANC visits that would further facilitate institutional deliveries. Whereas women from rural areas, poor economic status and lack of ANC visits were more likely to be associated with home delivery. However, this inequity in utilizing facility delivery decreases noticeably due to several initiatives taken by the governments and stakeholders providing financial and other incentives and establishment of rural birth centers to reduce home delivery [12–14]. However, still many women deliver at home in South-Asian countries and also without any SBAs during delivery. This might be due to cultural values and norms, religion, personal attitudes to facility birth, doctors and practitioners attitude towards women, waiting times, transport facilities and cost [35–37].

To increase facility delivery by women, Maharashtra India implemented incentives such as free services for facility delivery under the National Rural Health Mission (NRHM) program in 2006, which led to a substantial rise of (42% to 69%) facility delivery in Maharastra [38]. In rural areas of Pakistan, primary health care services were extended through deploying Lady Health Workers (LHWs), who provided MNCH services through regular home visits in rural areas [13]. In Bangladesh, the government deployed Family Welfare Visitors (FWVs), and Community Skilled Birth Attendants (CSBAs), Female Community Health Workers (FCHWs) in rural areas to provide basic maternity care and counseling and to encourage women to have facility delivery [12]. Likewise, In 2004, Nepal endorsed National Neonatal Health Strategy. Nepal’s Safe Motherhood and Newborn Health Long Term Plan 2006 to 2017 is guided by this strategy—this plan further emphasizes the inseparable nature of quality of health care during delivery and perinatal survival. Likewise, a policy on skilled birth attendants (SBAs) was endorsed in Nepal in 2006 to ensure SBA attendance at every childbirth [14]. The National Safe Motherhood Program has taken several initiatives to offer free delivery care and incentives for transportation to the healthcare facility for delivery [39]. In addition, healthcare facilities were subsidized for providing free delivery care based on the number of deliveries conducted in those facilities.

The overall prevalence or the proportion of women who had utilized health facility delivery is quite dissimilar across the three countries. Bangladeshi women had the lowest rate of health facility delivery (40%) while the rates were higher among Nepalese (62%) and Pakistani women (69%). Evidence shows that irrespective of home or health facility birth, attendance by an SBA is nearly universal in developed countries [40, 41]. However, in poor resource-setting countries like Bangladesh, Nepal and Pakistan, this has not yet been possible and often women are bound to give birth without an SBA or a midwife [6, 10, 15]. Childbearing is a precious life event for women [42], therefore choosing an appropriate and safe delivery place is vital to ensure a healthy mother and newborn and reduce maternal deaths and morbidities [6, 19, 43].

Findings from this study align well with those from previous studies. This study indicated that higher ANC visit is a strong predictor for facility delivery [6, 9, 10, 15, 19, 20]. During ANC visits women go through various counseling sessions about the importance of safe delivery practices and early detection of pregnancy and delivery complications, which might inspire them to prefer health facilities for delivery. This study showed that more younger women utilized facility delivery than older women, which however, does not correspond with the previous study findings in Bangladesh [10, 17, 44]. This association is possible since younger women in Bangladesh are now more educated and well aware of the risks and potential pregnancy complications that might influence their facility delivery decisions [10, 20]. In contrast, older women from Nepal and Pakistan were more likely to use facility delivery—an area that future studies in these countries could further investigate.

In this study, watching TV among women was found to be a facilitating factor for facility delivery. This is probably due to the fact that mothers watching TVs were more likely to receive updated maternal nutrition and child health (MNCH)and related information and hence potentially improve awareness on the importance of facility delivery [9].

Women’s education plays an important role in their decision-making power and economic solvency that influence their preferences for the place of delivery as educated women are more likely to be aware of the importance of regular antenatal check-ups and increased ANC visits. However, this did not hold true even among the three study countries. In Pakistan, non-educated women (39.9%) had a higher level of facility delivery than educated women, possibly due to various incentives offered to this group of women by various NGOs and similar institutions.

Husband’s decision-making power is a strong predictor influencing health facility delivery in Pakistan supported by previous studies [10, 45]. This might be due to men dominating society where women need their husbands’ approval to deliver in a health facility. However, men mostly in South-Asian communities are not actively involved in pregnancy and childbirth affairs considering pregnancy and childbirths as women’s only jobs [10, 46]. With this, women might end up giving birth at home, thus following the trajectory of their mothers or mothers-in-law. Often husbands are solely responsible for family income and therefore hold the dominant role in deciding on women’s healthcare and place of delivery. This is mostly due to the existing cultural differences, religion, personal beliefs that may cause inequity in decision-making power in this region [36, 39]. However, some studies reported that joined decision or effective communication between spouses, instead of independent decision influence the higher use of facility delivery [9].

Working women had lower odds of utilizing facility delivery which is in contrast to previous studies where working women had higher autonomy and hence have more decision making power on reproductive care and ANC visits [9, 10] that lead to facility delivery. In addition, working women have more economic solvency that helps cover facility delivery costs. Therefore, it is not mere education alone; women empowerment and economic solvency are of paramount importance in enhancing facility delivery and improving women’s health.

The main strengths of this study are that it utilized the latest demographic data from three similar developing countries in the South-Asian region using similar protocols. The sample size for each data set is immense, as the surveys were conducted at the national population level in each country. One major limitation of this study is that the DHS surveys are cross-sectional in design where both exposure/predictors and outcomes are measured at the same time-point and thus, no causal relationships can therefore be inferred. In addition, some explanatory variables were excluded from the model due to the unavailability of data. For example, we excluded distance, waiting time, the healthcare practitioners’ behavior, and availability of transportation facilities that might influence the study findings.

## Conclusions

This study suggests that both women and their husbands’ educational status, household economic status, and ANC visits were the key factors that influenced the place of delivery in three selected countries of South-Asia. Public health policies and interventions targeting availability and accessibility of birth centers, training and deployment of SBAs, use of mass media for health education and raising awareness, compulsory female education, the involvement of men in pregnancy and childbirth events, and providing financial incentives and subsidies to promote antenatal visits and facility delivery may encourage women in these countries to deliver at health facilities.
